# Buffalo Dispensaries

**Published:** 1870-09

**Authors:** 


### Buffalo Dispensaries.

There seems to be an active competition in the Dispensary business in Buffalo, a new competing line being now under consideration. If the entire medical business of the city should hereafter be conducted through this system, it would not be so great an innovation as might at first thought be supposed.

All our profession have, during the entire history of the town, kept open, at all hours of the day and night, a "free dispensary," where all who asked it were gratuitously prescribed for and carefully attended; and most of the members of our profession have furnished gratuitously from the present dispensary all medicines required by patients asking to receive them. Poor people have been, and now are, able to select their own physician, and receive both his services and necessary medicines gratuitously. We cannot see what further advantages they can desire, unless they are to receive pay for taking the medicines when thus furnished. Neither can we see any adequate advantages to be received by physicians, since patients who obtain the services of physicians gratuitously, when poor, when fortune changes avoid them, and prefer others who have not known them in the time of want. Indeed patients rarely prize services for which they pay nothing; they generally regard them as worthless. Physicians sometimes complain of their lot, and represent the business as unremunerative, arduous and thankless. True and strange if it were otherwise. Our charities are too numerous. We value our services at too low rate; we give our advice in many instances to those who, rather than follow it, go elsewhere and pay liberally for that which is really worth less.

We would suggest the indefinite expansion of our present institution, and an addition to its medical staff of all physicians willing to serve, with offices, at numerous stations, and urgent invitations to all to call early and take advice.

